# Stress, loneliness, depression, anxiety and problematic smartphone use among a sample of Syrian refugee adolescents: a network approach

**DOI:** 10.1186/s13034-025-00936-y

**Published:** 2025-06-18

**Authors:** Onat Yetim, Lut Tamam, Ayşegül Efe, İlham Sebea Alleil, Resul Çakır

**Affiliations:** 1 Psychology Department, Toros University Bahçelievler District, Mersin, Turkey; 2 Psychiatry Department, Çukurova University Balcalı Campus, Adana, Turkey; 3 Child and Adolescent Psychiatry Department, Etlik City Hospital, University of Health Sciences, Ankara, Turkey

**Keywords:** Syrian refugee adolescent, Stress, Loneliness, Depression, Anxiety, Problematic smartphone use

## Abstract

**Background:**

Previous studies have demonstrated the existence of complex associations between stress, loneliness, depression, anxiety, and smartphone addiction in adolescents. However, the paucity of studies evaluating the relevant relationships in migrant adolescents demands the elimination of uncertainty in a sample of adolescents exposed to trauma and chronic stressors.

**Method:**

This study utilizes network analysis to identify the central factors and potential bridging paths among these variables. Identifying central symptom clusters contributing to the maintenance of the overall network provides potential targets for clinical or policy-level interventions. Network analysis also enables a more nuanced understanding of how internalizing symptoms and behavioral dysregulation co-occur and reinforce one another. Employing 836 Syrian refugee adolescents, we obtained a stable network of the above variables.

**Results:**

Our results revealed that problematic smartphone use (PSU) was the most central node in the network. Both generalized anxiety disorder (GAD) and panic disorder also emerged as central nodes, reinforcing their transdiagnostic relevance. Bridge expected influence analysis revealed that stress, GAD, and PSU were key connectors between symptom domains.

**Conclusion:**

Our findings indicate that PSU may not simply be a byproduct of emotional problems but a key component of psychopathology in refugee adolescents. Notably, PSU exhibited strong partial correlations with GAD and social anxiety disorder, supporting prior research that links maladaptive smartphone use to emotional dysregulation and anxiety-related avoidance. Future research investigating protective factors, such as family support, peer support, and cultural integration, in the correlations between PSU and psychopathologies will lead to the development of effective interventions.

## Introduction

As a result of the ongoing civil war in Syria for over a decade, millions of Syrians have been forced to flee their homes. Turkey has emerged as the primary destination for Syrians seeking refuge, hosting 3,648,983 individuals [1, 2]. Most of this population comprises children and adolescents [3, 4]. While displacement affects the entire family, adolescents are susceptible to migration-related challenges due to their developmental stage, often attaching greater emotional significance to the experience than adults [5, 6].

Adolescents who are forced to migrate may encounter multiple stressors related to relocation [7], acculturation [8], and adaptation to new educational environments [9]. These are often compounded by family trauma, differences in school systems, and language barriers in the host country, all of which intensify the challenges faced by refugee youth [10–12]. Al-Shatanawi et al. [13] highlight social isolation and loneliness as prominent psychiatric difficulties among Syrian refugee adolescents. Loneliness, in particular, has been associated with impaired developmental and emotional outcomes during adolescence [14, 15]. Extensive research has documented inverse relationships between social support and internalizing symptoms such as depression and anxiety [16, 17]. In both Turkish and international contexts, numerous studies report that at least half of Syrian refugee adolescents experience symptoms of anxiety [18–21]. Furthermore, comparative studies reveal that refugee adolescents exhibit significantly higher levels of anxiety and depression than their non-refugee peers [22–26].

Loneliness, a core psychological construct in refugee populations, is strongly linked to both anxiety and depression [14, 15]. Defined as the perceived gap between desired and actual social relationships [27], it can intensify emotional vulnerability and social isolation. Syrian refugee adolescents, who often face linguistic and cultural obstacles in forming peer relationships, are especially susceptible to chronic loneliness [13, 28, 29].

In parallel, problematic smartphone use (PSU)—often conceptualized as smartphone addiction—has become a growing behavioral concern among adolescents worldwide [30, 31]. PSU is characterized by excessive and poorly regulated smartphone use that disrupts daily functioning, including academic performance, interpersonal relationships, and emotional well-being [32]. A growing body of research indicates that PSU is associated with elevated levels of stress, depression, anxiety, and sleep disturbances [33]. Many adolescents turn to smartphones as a means of coping with boredom, distress, or loneliness, leading to a self-reinforcing cycle of emotional dysregulation and behavioral dependence [34]. This maladaptive use, marked by poor emotional regulation, habitual checking behaviors, and high reward sensitivity, has been described as a form of behavioral addiction [35].

Moreover, PSU has been linked to decreased face-to-face interactions and weakened peer bonding [34], which may further exacerbate loneliness and emotional isolation. Although smartphones are intended to enhance social connectivity, excessive and emotionally driven usage may paradoxically intensify disconnection [28]. These concerns are particularly acute in high-risk populations such as refugee adolescents. A recent large-scale epidemiological study found that nearly one in five adolescents displayed problematic smartphone reliance, which was significantly associated with symptoms of depression and anxiety [36]. Despite growing scholarly attention to PSU, few studies have explored its psychological correlates in forcibly displaced youth, underscoring the need for targeted research in this area.

Stress—particularly perceived—has consistently been identified as a critical predictor of PSU and internalizing disorders [37–39]. Adolescents experiencing elevated stress levels are more likely to use smartphones as an avoidance strategy [40, 41]. Furthermore, chronic stress exposure is thought to impair emotional regulation, thereby increasing susceptibility to PSU and reinforcing maladaptive coping cycles [42, 43]. Among refugee adolescents, ongoing exposure to both trauma-related and post-migration stressors may make this link especially salient [44, 45].

In light of these interrelated challenges, recent theoretical models in psychopathology have emphasized that mental health disorders emerge from dynamic interactions among symptoms rather than from singular latent causes [46]. The network approach operationalizes this perspective by conceptualizing psychological constructs as nodes in a system of mutually reinforcing relationships, whereby the activation of one symptom (e.g., perceived stress) may influence others (e.g., negative mood or behavioral dysregulation) [47]. This model challenges traditional reliance on total scale scores or diagnostic categories, which may obscure the functional relationships between individual symptoms [48].

Given the complex and cumulative nature of psychosocial risk factors experienced by refugee adolescents—such as trauma exposure, acculturation stress, peer isolation, and limited coping resources—traditional variable-based models may fall short in capturing the intricate interdependencies among psychological symptoms in this population. Constructs like stress, loneliness, depression, and anxiety may not function as isolated predictors of problematic outcomes such as PSU; instead, they likely interact in dynamic ways, with their influence emerging through interconnected patterns. Network analysis offers a robust framework for examining these relationships by identifying central nodes (e.g., generalized anxiety) and bridge nodes (e.g., stress), thus enabling a more nuanced understanding of how internalizing symptoms and behavioral dysregulation co-occur and reinforce one another. This approach provides a data-driven basis for developing targeted clinical or policy-level interventions [49, 50].

Specifically, nodes with high centrality are referred to as central symptoms. In terms of psychopathology, central symptoms or symptom clusters are associated with many other symptoms and are potentially critical to the development of the whole network. Regarding the clinical implications, central nodes are crucial for activating or inhibiting other nodes and significantly contribute to the maintenance of the overall network [49, 50]. Interventions targeting central nodes could disrupt the entire network, facilitating treatment and prevention.

Nodes connecting these core variables are referred to as bridging symptoms that are critical to maintaining the co-occurrence of variables and transmitting the influence of one variable on another [50]. Bridging symptoms facilitates the development of comorbidities or intricate connections between different psychopathologies. The network graph consists of interconnected nodes with edges that illustrate the presence of a relationship between nodes, with the thickness of the edges representing the intensity of the connection.

Previous studies have investigated the network structure of PSU and its associated variables in various adolescent populations [29, 51, 52]. However, to date, no study has examined a dimension-level network incorporating loneliness, stress, anxiety, depression, and PSU within a refugee adolescent sample. This gap limits our understanding of how these psychological dimensions interact in contexts marked by forced displacement and cumulative psychosocial adversity.

The present study aimed to investigate the network structure of stress, loneliness, depression, anxiety, and PSU in a sample of Syrian refugee adolescents. Specifically, we sought to identify the most central and bridging nodes within this network to better understand how internalizing symptoms and behavioral dysregulation interact in this population. By applying network analysis to data collected from adolescents exposed to chronic displacement-related adversity, we aimed to uncover symptom-level pathways that sustain PSU and offer insight into potential targets for tailored clinical or policy-level interventions.

## Methods

### Participants and procedure

The study sample consisted of 836 Syrian refugee adolescents (56.1% female) aged between 12 and 18 years (M = 14.20, SD = 2.07) living in various provinces of Turkey. The Syrian people were forced to migrate following the outbreak of civil war. 2011 marks the beginning of migration to Turkey. All the youths participating in our study have been living in Turkey for a maximum of 12 years, with an average of 8.4 years.

An exploratory quantitative research design guided the cross-sectional sampling method to recruit adolescents. Participants were recruited from schools and community centers in collaboration with local NGOs and school administrations, relying on convenience sampling. The inclusion criteria required participants to be of Syrian origin, aged 12–18, and enrolled in formal or non-formal education programs in Turkey.

Participants who were literate in their native language and volunteered to participate in the study were included in the data collection process. Considering the recommendations of Tabachnick et al. [53] for calculating the sample size based on the number of scale items, 917 adolescents who agreed to participate in the study were included. After invalid data was checked, the number of participants decreased to 836. During the data collection process, either one of the researchers or the psychological counselors from formal or non-formal education programs supervised the participants, addressing their possible problems by responding to their queries and concerns.

The Turkish Statistical Institute (TÜİK), Turkey's official statistics agency, publishes data on household income satisfaction rates every year [54]. Here, household income levels are evaluated in five separate groups based on individual satisfaction. These groups are divided into very satisfied, satisfied, neither satisfied nor dissatisfied, dissatisfied, and very dissatisfied. In our study, an item was created for the satisfaction question, and a Likert-type scale was used to evaluate the participants' responses. According to TÜİK data for 2023, the percentages of "very dissatisfied," "dissatisfied," "neither satisfied nor dissatisfied," "satisfied," and "very satisfied" across Turkey were 8.2%, 30.1%, 24.1%, 36.1%, and 1.5%, respectively [55]. In our study, the percentages of "very dissatisfied," "dissatisfied," "neither satisfied nor dissatisfied," "satisfied," and "very satisfied" were 2.5%, 14.8%, 42.5%, 24.3%, and 15.8%, respectively.

These responses only reflect satisfaction rates and do not include an objective assessment of socioeconomic status. It is thought that Syrian participants, who may not want to reflect on their adverse economic circumstances, may have preferred a more neutral expression—"neither satisfied nor dissatisfied." This interpretation may explain the differences in the rates compared to TÜİK data. In addition to the demographic characteristics of the participants, items were created regarding the amount of time they spent on their smartphones and the frequency with which they checked their smartphones per day. The participants themselves rated these items.

Table 1 presents the participants' demographic and smartphone usage characteristics. Participation was voluntary, and written informed consent was obtained from all adolescents and their parents or legal guardians before data collection. The study was approved by the ethical committee of [Anonymized Institution]. Data collection was performed between February and April 2024.

**Table 1:** Sample characteristics

Variables	Groups	*n*	*%*
Gender	Female	469	56.1
	Male	367	43.9
Attendance status	Attending	774	93.1
	Not Attending	54	6.9
Age	12	250	31.0
	13	118	14.6
	14	112	13.9
	15	87	10.8
	16	83	10.3
	17	53	6.6
	18	103	12.8
Household income satisfaction	Very dissatisfied	21	2.5
	Dissatisfied	123	14.8
	Neutral	353	42.5
	Satisfied	202	24.3
	Very satisfied	131	15.8
Number of smartphone checks per day	< 10	210	26.0
	10–20	252	31.2
	20–30	137	17.0
	30–40	94	11.6
	> 40	115	14.2
Time spent on a smartphone per day	< 1 h	98	11.9
	1–2 h	247	30.0
	2–3 h	176	21.4
	3–4 h	107	13.0
	4–5 h	91	11.1
	5–6 h	39	4.7
	> 6 h	65	7.9
Employment status	Employed	109	13.3
	Unemployed	724	86.9
Turkish proficiency	Low proficiency	45	5.4
	Moderate proficiency	301	36.2
	High proficiency	485	58.4

Attendance status displays whether youths are continuing their formal education at schools

## Measurements

### Screen for child anxiety related disorders (SCARED)

To assess anxiety symptoms, we used the 41-item SCARED [56], which assesses five anxiety dimensions: panic disorder, generalized anxiety disorder, separation anxiety disorder, social anxiety disorder, and school avoidance. Items are rated on a 3-point Likert scale (0 = Not true, 1 = Sometimes true, 2 = Very true or often true), and total scores range from 0 to 82. A score of 25 or higher suggests the presence of clinically relevant anxiety. The Arabic version adapted by Hariz et al. [57] showed good internal consistency in this study (α = 0.86). In the current study, McDonald's omega (ω) value for SCARED was calculated to be 0.86.

### Kutcher adolescent depression scale—6 (KADS-6)

To asses depression symptoms, we used the 6-item Kutcher Adolescent Depression Scale (KADS-6) [58]. Items are rated on a 4-point Likert scale (0 = Hardly Ever to 3 = All of the Time), and total scores range from 0 to 18. Higher scores indicate greater severity of depression. The Arabic version adapted by Al-Modallal et al. [59] showed good internal consistency in this study (α = 0.80). In the current study, McDonald's omega (ω) value for KADS-6 was calculated to be 0.81.

### UCLA loneliness scale—6 (ULS-6)

To assess perceived loneliness, we used the 6-item UCLA Loneliness Scale (ULS-6) [60]. Items are rated on a 4-point Likert scale (1 = Never to 4 = Often), with total scores ranging from 6 to 24. Higher scores reflect greater perceived loneliness. The Arabic version adapted by Al Khatib [61] showed good internal consistency in this study (α = 0.79). In the current study, McDonald's omega (ω) value for ULS-6 was calculated to be 0.80.

### Perceived stress scale—10 (PSS-10)

To assess perceived stress, we used the 10-item Perceived Stress Scale (PSS-10) [62]. Items are rated on a 5-point Likert scale (0 = Never to 4 = Very Often). The total score ranges from 0 to 40, with higher scores indicating greater perceived stress. The Arabic version adapted by Chaaya et al. [63] showed good internal consistency in this study (α = 0.77). In the current study, McDonald's omega (ω) value for PSS-10 was calculated to be 0.76.

### Smartphone addiction scale—short version (SAS-SV)

To assess problematic smartphone, we used the 10-item Smartphone Addiction Scale—Short Version (SAS-SV) [64]. Items are rated on a 6-point Likert scale (1 = Strongly Disagree to 6 = Strongly Agree), and total scores range from 10 to 60. Higher scores reflect a greater risk of smartphone addiction. The Arabic version adapted by Hawi and Samaha [65] showed good internal consistency in this study (α = 0.88). In the current study, McDonald's omega (ω) value for SAS-SV was calculated to be 0.85.

### Statistical analysis

Statistical analyses were conducted using SPSS (Version 26.0) and R (Version 4.4.0) within RStudio. Before analysis, missing values were examined. Because less than 5% of the data were missing and Little’s MCAR test indicated that the data were missing completely at random (MCAR), we used mean imputation to replace missing values with the variable’s mean score. Mean imputation is considered a practical and minimally biased approach under MCAR conditions when the extent of missingness is low [66]. The reliability of the scale scores was evaluated using McDonald’s omega (ω), which is considered a more accurate internal consistency estimation than Cronbach’s alpha, particularly when factor loadings vary across items [67].

Network analysis was employed to examine the relationships among stress, loneliness, depression, anxiety subdimensions, and problematic smartphone use. We estimated the network using the Graphical Gaussian Model (GGM) in conjunction with the Extended Bayesian Information Criterion graphical least absolute shrinkage and selection operator (EBICglasso) algorithm. This regularized partial correlation model produces sparse, interpretable networks by penalizing weak associations and reducing false positive edges [68]. Network estimation and visualization were performed using the qgraph package in R [69].

In the estimated network, nodes represented psychological variables or symptom dimensions, while edges represented regularized partial correlations between them. Blue edges indicated positive associations and red edges indicated negative associations. Edge thickness corresponded to the strength of the relationship.

To assess the importance of nodes, we calculated Expected Influence (EI), which captures both the strength and direction of a node’s connections. EI is particularly suitable for networks with both positive and negative edges and is more stable than traditional centrality metrics such as betweenness and closeness [70, 71]. Centrality indices were visualized using the centralityPlot and centralityTable functions within qgraph [69]. In addition to EI, we calculated Bridge Expected Influence (bEI) using the networktools package to identify bridge nodes—variables that connect distinct symptom communities [72]. Identifying such nodes is valuable for clinical intervention, as targeting bridge nodes may reduce comorbidity between domains (e.g., stress and smartphone addiction).

To assess network stability and accuracy, we used the bootnet package [68]. Non-parametric bootstrapping with 10,000 iterations generated confidence intervals for edge weights. The correlation stability coefficient (CS-coefficient) was computed via case-dropping bootstrap, which evaluates how centrality estimates fluctuate when sample subsets are randomly removed. According to Epskamp et al. [68], a CS-coefficient above 0.25 is acceptable, and a value above 0.50 is preferred for stable interpretation of centrality metrics.

## Results

### Network estimations

**Fig. 1:**
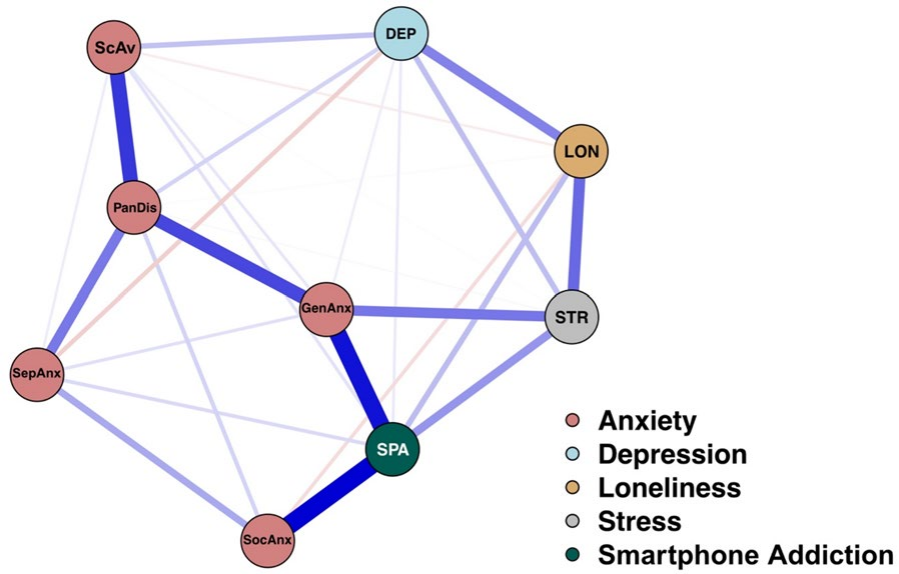
Estimated network structure of stress, loneliness, depression, anxiety subdimensions, and smartphone addiction. Edge thickness indicates the strength of the association, while blue edges indicate positive correlations and red edges indicate negative correlations. *ScAv* School avoidance, *PanDis* Panic disorder, *SepAnx* Seperation anxiety, *SocAnx* Social anxiety, *GenAnx* Generalized anxiety, *STR* Stress, *SPA* Smartphone addiction, *Lon* Loneliness, *Dep*x Depression

The estimated network structure revealed predominantly positive partial correlations among the assessed variables. Notably, the strongest associations emerged between smartphone addiction and social anxiety disorder (r = 0.46), smartphone addiction and generalized anxiety disorder (r = 0.43), school avoidance and panic disorder (r = 0.36). These findings suggest that problematic smartphone use may be particularly intertwined with specific anxiety subdimensions, especially those involving social discomfort and generalized worry. In contrast, weaker associations were observed between other variables—such as separation anxiety and depression—indicating a more peripheral role for these symptoms in the overall network configuration. The visualized network structure is presented in Fig. 1.

**Fig. 2:**
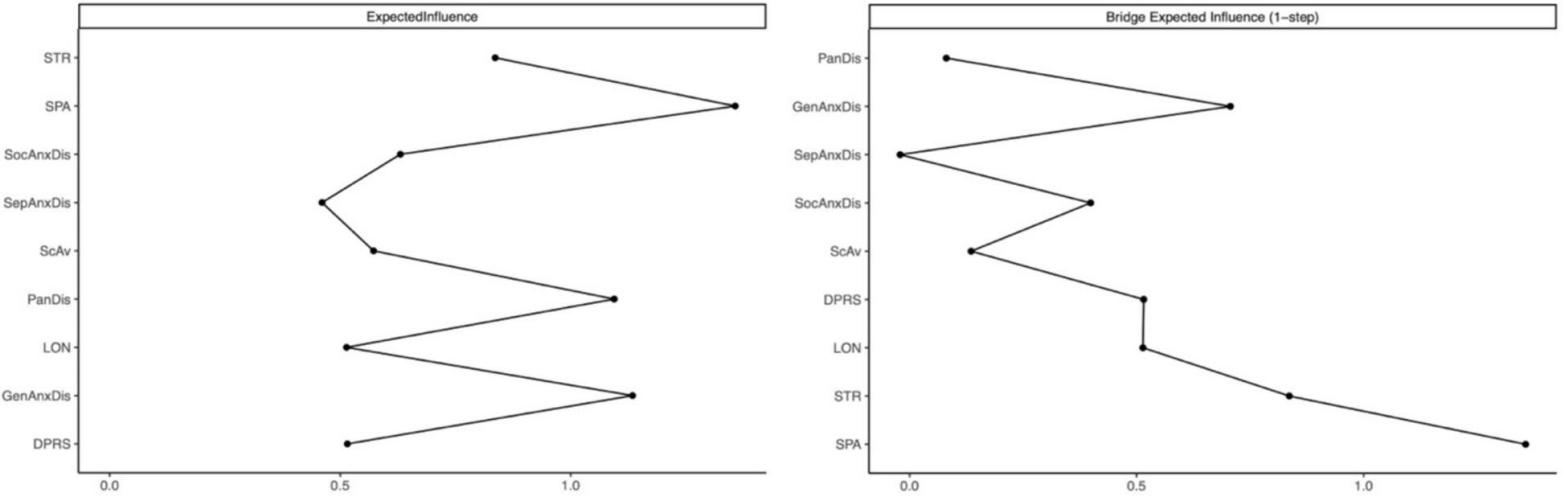
Expected influence (EI; left graph) and bridge expected influence (bEI; right graph) for every node. For each plot, *y* axis represent the nodes and *x* axis represent the EI and bEI. *ScAv* School avoidance, *PanDis* Panic disorder, *SepAnx* Seperation anxiety, *SocAnx* Social anxiety, *GenAnx* Generalized anxiety, *STR* Stress, *SPA* Smartphone addiction, *Lon* Loneliness, *Dep* Depression

To examine the relative importance of individual symptoms within the network, EI centrality scores were computed. The results showed that smartphone addiction (EI = 1.72) emerged as the most central symptom, followed by generalized anxiety disorder (EI = 1.04) and panic disorder (EI = 0.92). These nodes displayed strong connectivity across multiple psychological domains, highlighting their potential role in maintaining the network’s overall structure. In contrast, separation anxiety (EI =–1.00) and loneliness (EI =–0.84) had the lowest EI values, suggesting that these symptoms exert limited influence on the activation or maintenance of other network components. Interestingly, stress exhibited a modestly positive EI value (EI = 0.14), indicating a moderate degree of centrality within the network (see Fig. 2).

In addition to centrality, bridge expected influence (bEI) scores were calculated to identify nodes that connect distinct symptom communities. Results indicated that smartphone addiction was not only the most central node but also the most prominent bridge symptom (bEI = 1.36). Other key bridge nodes included stress (bEI = 0.84) and generalized anxiety disorder (bEI = 0.71), suggesting these symptoms serve as critical points of interaction between emotional distress and behavioral dysregulation. Further bridge connections were identified for depression (bEI = 0.52) and loneliness (bEI = 0.51), while separation anxiety (bEI =–0.02) had negligible bridging influence, reflecting its peripheral integration in the symptom network (see Fig. 2).

Finally, the stability of centrality estimates was assessed through non-parametric case-dropping bootstrapping. The correlation stability coefficient (CS-coefficient) for expected influence was 0.75, indicating excellent robustness and allowing for confident interpretation. The bridge expected influence index also yielded a CS-coefficient of 0.75, confirming its reliability.

## Discussion

This study investigated the network structure of internalizing symptoms, loneliness, stress, and PSU among Syrian refugee adolescents, offering a nuanced view of how these symptoms interact in a population exposed to chronic adversity. By applying network analysis, we identified central and bridge symptoms that likely contribute to the co-occurrence and maintenance of psychological distress in this group.

Our results revealed that PSU was the most central node in the network. This finding indicates that PSU may not simply be a byproduct of emotional problems but a key component of the psychopathology network in refugee adolescents. Notably, PSU exhibited strong partial correlations with generalized anxiety disorder (GAD) and social anxiety disorder, supporting prior research that links maladaptive smartphone use to emotional dysregulation and anxiety-related avoidance [28, 29, 73]. In vulnerable populations, such as adolescence with refugee history and ongoing post-migration stressors, smartphones have been found to serve as instruments for avoiding distressing emotions or unpleasant social contexts. However, excessive reliance on these devices may lead to an exacerbation of feelings of isolation and anxiety over time [74].

Both GAD and panic disorder also emerged as central nodes, reinforcing their transdiagnostic relevance. These psychopathologies were closely linked to other internalizing symptoms and stress, aligning with prior studies that report high prevalence rates of GAD and panic symptoms among refugee adolescents [75, 76]. Exposure to unpredictable environments, traumatic loss, and complex post-migration stressors may foster persistent worry, hypervigilance, and somatic arousal, all of which are characteristic of anxiety disorders. These findings highlight the need for intervention strategies that are tailored to address the deficits in emotional regulation and tolerance of uncertainty among young refugees.

Bridge expected influence analysis revealed that stress, GAD, and PSU were key connectors between symptom domains. Bridge nodes are significant for understanding comorbidity, as they facilitate the transmission of psychopathological influence across otherwise distinct symptom clusters. Notably, stress emerged as a prominent bridge between internalizing symptoms and PSU, supporting prior findings that link perceived stress with both emotional dysregulation and excessive smartphone use in adolescents [38, 40, 42]. In refugee adolescents, stress is likely intensified by chronic exposure to trauma, socioeconomic insecurity, and acculturation pressures [44], which can simultaneously trigger internalizing symptoms and promote maladaptive coping patterns.

Although loneliness was not among the most central symptoms, it demonstrated moderate bridge influence and a robust connection with depression. This is consistent with literature emphasizing the role of loneliness in the onset and maintenance of depressive symptoms, including suicidal ideation, during adolescence [74, 77]. Refugee adolescents often face prolonged experiences of cultural alienation and peer disconnection, which may exacerbate loneliness and heighten vulnerability to depression [13, 14]. Social integration and peer support programs may serve as vital protective factors against the development of more severe psychopathology [12, 21]. Depressive symptoms were found to be closely linked with loneliness in the network structure, highlighting the social-emotional dimension of depression in refugee adolescents. This supports prior findings suggesting that perceived social disconnection plays a critical role in the emergence of depressive symptoms [77, 78].

The overall structure of the symptom network points to several implications for clinical and policy-level action. Targeting central and bridge symptoms—particularly PSU, GAD, and stress—may effectively interrupt the feedback loops that perpetuate distress and dysfunction. Interventions that strengthen stress-coping abilities, improve emotional regulation, and promote healthier technology use may yield cascading benefits across the network. Additionally, enhancing peer connectedness, cultural integration, and identity support may help reduce feelings of loneliness and mitigate depressive trajectories. Such multi-level interventions are significant for refugee adolescents, who often lack access to traditional protective systems such as extended family networks or stable community structures [45].

## Limitations

This study has several limitations that should be taken into account when interpreting the findings. Firstly, measures of adolescent anxiety, depression, stress, loneliness, and smartphone addiction were only assessed through self-reporting. Self-reporting measures may be subject to response bias or social desirability bias. Therefore, the results should not be used as a substitute for direct assessments by mental health professionals, and caution should be exercised when interpreting the results. Secondly, the study's cross-sectional design does not allow us to conclude the direction of the relationships or the causal effects of the variables on each other. Therefore, future studies should use longitudinal designs to establish temporal relationships among variables and examine causal relationships.

Finally, our study was conducted with 836 migrant adolescents in three cities of Turkey through non-governmental organizations. There is a need for comprehensive studies, including those of other provinces of Turkey. Further research is also needed in other locations where Syrian refugee youths face different post-migration stressors, such as Western countries and Arabic-speaking countries like Jordan and Libanon.

## Conclusion

To the best of our knowledge, this study is the first to investigate the symptom-level interplay between stress, loneliness, problematic smartphone use, and internalizing symptoms in Syrian refugee adolescents using network analysis. By identifying both central and bridge symptoms, we demonstrated that perceived stress, generalized anxiety disorder, and problematic smartphone use occupy pivotal positions in the network, acting as key drivers of comorbidity and psychological distress. Our findings suggest that interventions targeting central symptoms—such as stress and generalized anxiety disorder—may yield widespread benefits across multiple domains of mental health. Furthermore, identifying bridge symptoms, including stress and loneliness, highlights the importance of addressing individual symptom clusters and pathways that link emotional and behavioral difficulties. Recognizing these connections may enhance early intervention efforts and guide the development of comprehensive, multi-level prevention strategies. While network analysis offers valuable insights into the structure of psychological symptoms, its application should be complemented by longitudinal and multi-informant studies to clarify further the temporal dynamics of symptom networks in high-risk populations. Overall, our results provide a foundation for understanding the complex interrelations among stress, loneliness, and behavioral addictions in refugee adolescents and point to actionable targets for future clinical and community-based interventions.

## Data Availability

No datasets were generated or analysed during the current study.
